# Going far beyond the near-field diffraction limit via plasmonic cavity lens with high spatial frequency spectrum off-axis illumination

**DOI:** 10.1038/srep15320

**Published:** 2015-10-19

**Authors:** Zeyu Zhao, Yunfei Luo, Wei Zhang, Changtao Wang, Ping Gao, Yanqin Wang, Mingbo Pu, Na Yao, Chengwei Zhao, Xiangang Luo

**Affiliations:** 1 State Key Laboratory of Optical Technologies on Nano-fabrication and Micro-engineering, Institute of Optics and Electronics, Chinese Academy of Sciences, P.O. Box 350, Chengdu 610209, China

## Abstract

For near-field imaging optics, minimum resolvable feature size is highly constrained by the near-field diffraction limit associated with the illumination light wavelength and the air distance between the imaging devices and objects. In this study, a plasmonic cavity lens composed of Ag-photoresist-Ag form incorporating high spatial frequency spectrum off-axis illumination (OAI) is proposed to realize deep subwavelength imaging far beyond the near-field diffraction limit. This approach benefits from the resonance effect of the plasmonic cavity lens and the wavevector shifting behavior via OAI, which remarkably enhances the object’s subwavelength information and damps negative imaging contribution from the longitudinal electric field component in imaging region. Experimental images of well resolved 60-nm half-pitch patterns under 365-nm ultra-violet light are demonstrated at air distance of 80 nm between the mask patterns and plasmonic cavity lens, approximately four-fold longer than that in the conventional near-field lithography and superlens scheme. The ultimate air distance for the 60-nm half-pitch object could be theoretically extended to 120 nm. Moreover, two-dimensional L-shape patterns and deep subwavelength patterns are illustrated via simulations and experiments. This study promises the significant potential to make plasmonic lithography as a practical, cost-effective, simple and parallel nano-fabrication approach.

For conventional far-field optics, the imaging resolution is diffraction limited and cannot exceed approximately half of the wavelength of light^1^. However, in near-field imaging, evanescent waves delivering subwavelength information have been used to achieve subdiffraction imaging in both scanning near-field optical microscopes (SNOMs)^2^,^3^ and near-field lithography^4^,^5^,^6^,^7^. Unfortunately, near-field imaging suffers from the near-field diffraction limit, in which the resolvable feature of an object is strongly constrained by the illumination light wavelength and the air distance between the imaging devices and objects^8^,^9^. Therefore, SNOM requires fiber tip in close proximity to the sample surface, and the mask patterns are often physically contacted with the recording photoresist (Pr) layer.

In 2000, Pendry first proposed the concept of superlens, in which subwavelength imaging could be obtained by amplifying evanescent waves through a metallic film, owing to surface plasmon excitation^1^. In 2004, Luo reported that deep subwavelength surface plasmons interference pattern with 50-nm feature size was successfully recorded by Pr below a Ag film with grating structures^10^. In 2005, a superlens of Ag film at the I-line 365-nm wavelength was experimentally demonstrated with a 60-nm half-pitch resolution^11^. Subsequently, further theoretical and experimental investigations were performed to improve resolution, fidelity and pattern aspect ratio of superlens imaging, for example by use of a smooth Ag lens^12^,^13^,^14^,^15^,^16^,^17^, a plasmonic reflection lens^18^,^19^,^20^, or a loss reduction superlens^21^,^22^. Despite those superresolution methods, the most formidable obstacle associated with the near-field diffraction limit has not been effectively addressed. The distance between the object and superlens is extreme small so that nearly physical contact mode is employed in all of the experimental demonstrations^10^,^11^,^12^,^13^,^17^,^18^,^19^,^20^,^21^. This physical contact hampers the promising applications of these superlens approaches in practical lithography, owing to the scratching and abrasion of the mask patterns, among other concerns.

To address these concerns, efforts are underway to develop novel methods for the control and measurement of air distances in a few tens of nanometers, such as air-bearing sliders used in flying plasmonic lens lithography^23^, and white-light scanning interferometers^24^,^25^,^26^,^27^. Achieving such a method would promote the non-contacted mode application of a plasmonic lens in the near-field regime and facilitate the increase of the air working distance of near-field lenses, which is widely acknowledged as a significant challenge.

In this study, plasmonic cavity lens with high spatial frequency spectrum off-axis illumination (OAI) is applied to address the short working distance issue of near-field diffraction-limited imaging. Both experimental and theoretical demonstrations were performed to show that the imaging resolution and contrast could be greatly enhanced in the case of a large air distance between the object patterns and plasmonic cavity lens. The approach promises to improve the performance of non-contact plasmonic cavity lens lithography, with an air distance approximately 4–6 times longer than that of conventional near-field photolithography.

## Results

### Configuration and simulations

**Figure 1:**
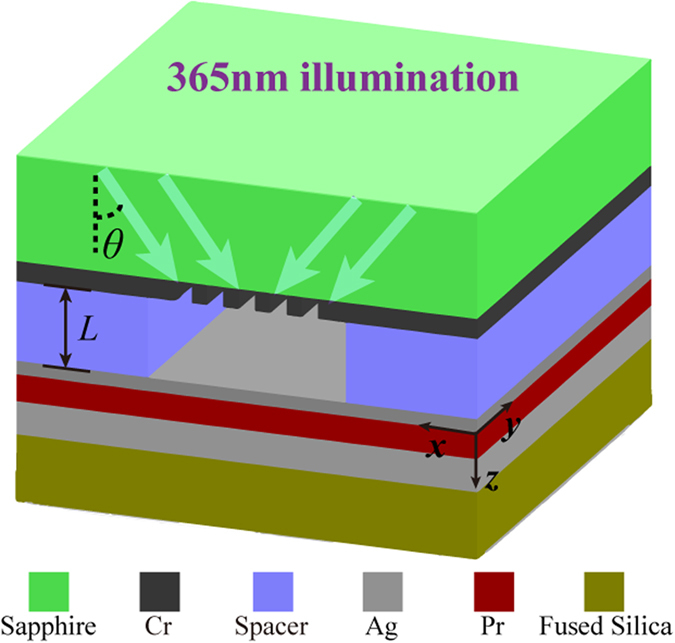
Schematic configuration of plasmonic cavity lens imaging under high spatial frequency spectrum OAI used to exceed the near-field diffraction limit.

Figure 1 shows a schematic configuration of the subwavelength imaging with a plasmonic cavity lens and high spatial frequency spectrum OAI. Ultraviolet light at the central wavelength 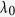 of 365 nm from the I-line of a mercury lamp source impinges the 40-nm-thick Cr mask patterns in an off-axis manner to generate the high spatial frequency spectrum illumination beam with the incidence transverse wavevector 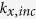 being 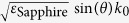, where 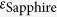 is the permittivity of sapphire substrate; *θ* is the light incidence angle and *k*_0_ = 2*π*/*λ*_0_ is the wavevector of light in vacuum. The incidence plane of two symmetrical illumination lights is perpendicular to the line patterns. To generate an air gap between patterns and plasmonic cavity lens in lithography experiments, a Cr spacer is placed around the mask pattern region. The plasmonic cavity lens structure fabricated on a fused silica substrate, which is composed of a 20-nm-thick top Ag layer, a 30-nm-thick Pr layer and a 50-nm-thick bottom Ag layer, is located below the mask. In the imaging process, the thickness of the Cr spacer layer determines the air distance because the Cr mask is in physical contact with the plasmonic cavity lens via air pressure. The permittivities for the used materials at the wavelength of light of 365 nm are 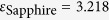
28, 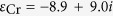
28, 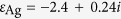
29, and 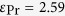
11.

**Figure 2:**
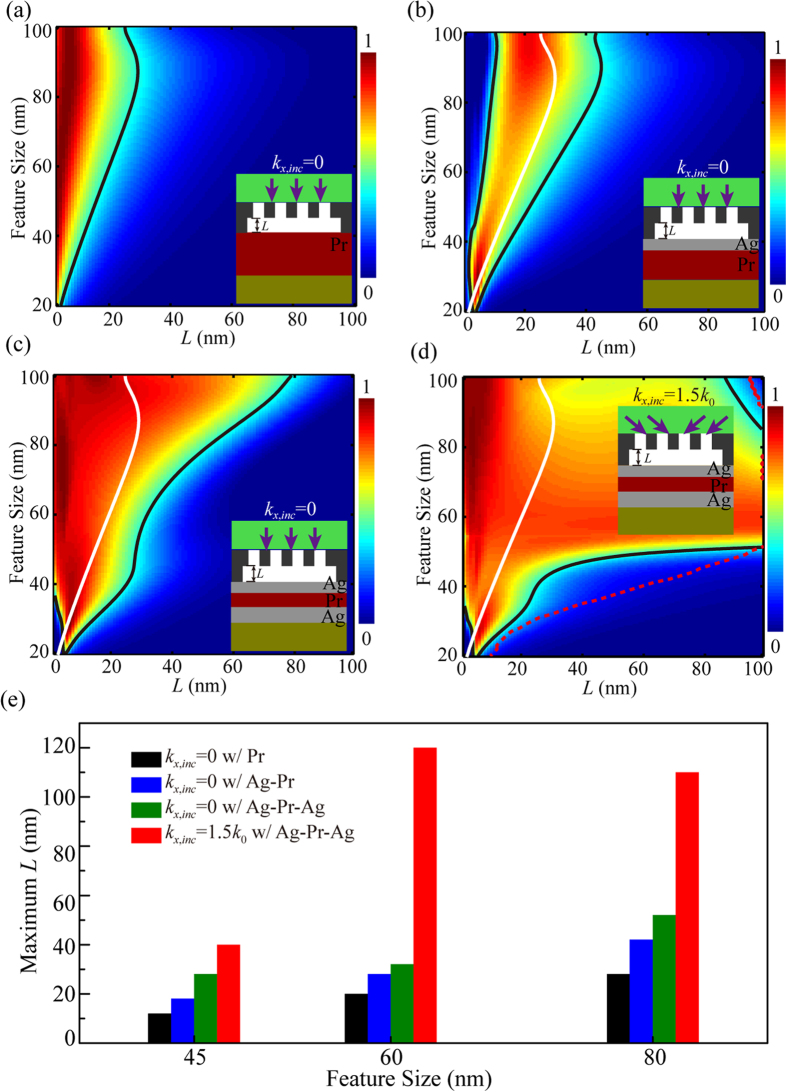
Imaging contrast as a function of the air distance (*L*) and half-pitch size of dense-line object for (a) the near-field imaging under normal incidence (NI, *k*_*x*,*inc*_ = 0), (b) the superlens under NI, (c) the plasmonic cavity lens under NI and (d) the plasmonic cavity lens under high spatial frequency spectrum OAI (*k*_*x*,*inc*_ = 1.5 *k*_0_) from two sides. The contrast contours of 0.4 are denoted by the black curves in (**a–d**). The white curves in (**b–d**) are replications of the black contour in (**a**), corresponding to the limit of the conventional near-field imaging. The red dashed curve in (**d**) corresponds to the results of the approximate model of Eq. 2 in the text. (**e**) Calculated maximum air distance for the four imaging schemes.

Figure 2a–d show the dependence of imaging contrast on air gap distance and half pitch of dense lines (L/S = 1) ranging from 20 to 100 nm for the near-field, superlens and plasmonic cavity lens imaging schemes with or without high spatial frequency spectrum OAI. As expected, a larger air distance corresponds to an increase in the feature size of the objects in all imaging schemes. To provide a qualitative evaluation and comparison in this study, the near-field diffraction limit criterion is defined by 0.4 contrast contour of the conventional near-field lithography as the black curve shown in Fig. 2a. For the conventional near-field imaging scheme with *k*_*x*,*inc*_ = 0 (Fig. 2a), large imaging contrast requires the image recording medium Pr proximity to the object’s surface. The results could be validated by considering that the field intensity of the evanescent waves from the object is reduced to 1/e = 0.37 of its original value within the characteristic decay length *L* in air surrounding media^11^


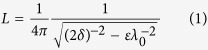


where *δ*, *L*, *λ*_0_ and *ε* are the half pitch of line-array object, the air distance, the wavelength of light in vacuum and the permittivity of air, respectively.

Regarding the superlens structure in Fig. 2b, surface plasmons excitation helps to amplify evanescent waves in the imaging region compared to the absence of the superlens^1^,^11^. However, the air working distance could not be significantly improved in comparison with the near-field diffraction limit curve in Fig. 2a. The superlens imaging fails to function as the air distance tends to zero. This phenomenon is attributed to the damped excitation of surface plasmon modes for the Ag superlens with nearly contacted metallic mask pattern. For the plasmonic cavity lens under normal incidence (NI) shown in Fig. 2c, the range of the air distance is further slightly widened, benefiting from the additional reflection enhancement of evanescent waves in the cavity lens imaging process^30^. In addition, the imaging quality is of high contrast in the region for air distance close to zero.

By adopting a plasmonic cavity lens with *k*_*x*,*inc*_ = 1.5 *k*_0_ illumination, as shown in Fig. 2d, high-contrast imaging is possible even for an air distance that greatly exceeds the near-field diffraction limit curve with the feature size of the object greater than 50 nm. For smaller feature-size patterns, the air distance extension is not obvious in this design and could be further improved by utilizing higher spatial frequency spectrum OAI light, as demonstrated below. The bar-shape graph in Fig. 2e plots the maximum air working distance for the four imaging schemes with feature-size objects of 45, 60 and 80 nm. The maximum air distance could reach 120 nm, approximately 6-fold larger than that of the conventional near-field imaging for 60-nm feature-size object.

### Principle and approximate model

**Figure 3:**
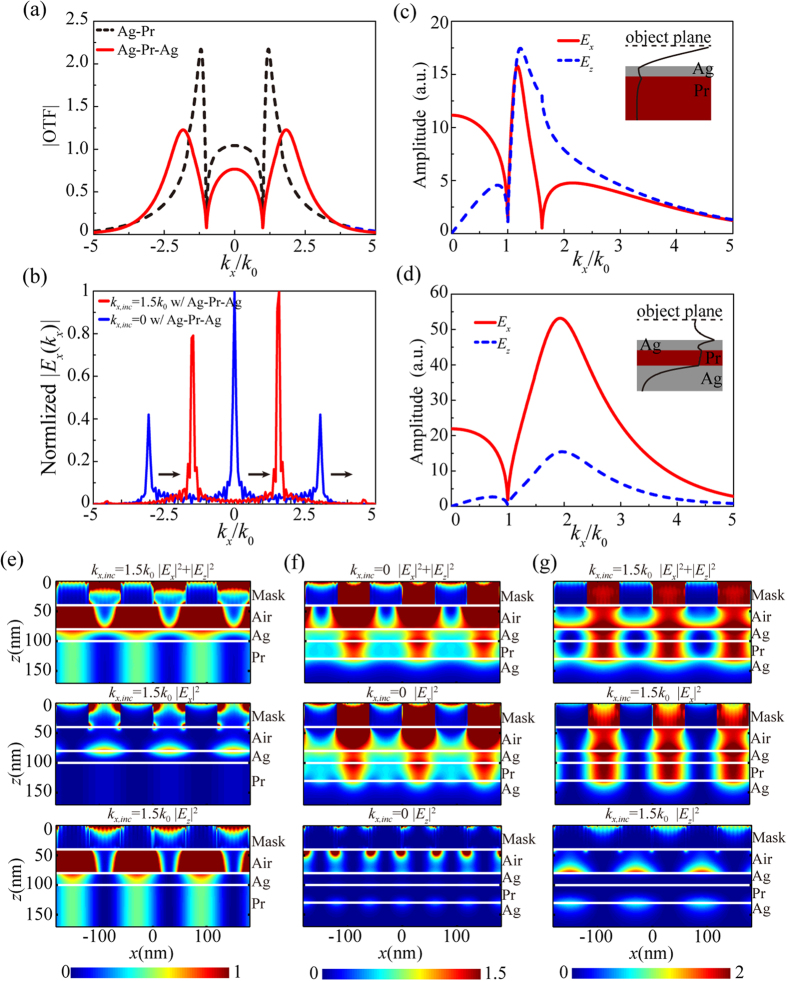
(**a**) Optical transfer function |OTF| in the measure of |*H*_*y*_| for the plasmonic cavity lens and superlens. (**b**) Fourier spectra of the image plane for the plasmonic cavity lens imaging under NI (*k*_*x,inc*_ = 0) and high spatial frequency spectrum OAI (*k*_*x,inc*_ = 1.5 *k*_0_) on one side. Transmission amplitude of the image plane in the measure of |*E*_*x*_| and |*E*_*z*_| for the superlens (**c**) and plasmonic cavity lens (**d**). The calculation plane is fixed at 15 nm away from the top surface of the Pr layer. The insets in (**c**) and (**d**) are the electric intensity distributions in the superlens and plasmonic cavity lens, respectively. Cross-sectional views of |*E*_*x*_|^2^+|*E*_*z*_|^2^, |*E*_*x*_|^2^, and |*E*_*z*_|^2^ for the imaging scheme with the superlens under high spatial frequency spectrum OAI (**e**) and the plasmonic cavity lens under NI (**f**) and high spatial frequency spectrum OAI (**g**). The air gap distance is 40 nm, and the half pitch of the mask patterns is 60 nm.

To gain insight into how the proposed method far exceeding the diffraction limit of near-field optics, further investigations were made by considering one-dimensional subwavelength dense-line patterns and analyzing the optical transfer functions (OTFs) in the imaging process. Compared with the superlens structure, a plasmonic cavity lens enables to transfer the evanescent waves in a wider wavevector range inside the Pr region, as the OTFs plotted in Fig. 3a. This phenomenon is mainly attributed to the surface plasmon excitations at the interface of the Pr and bottom Ag layer, which delivers the reflection enhancement of evanescent waves transmitted through the top Ag layer^30^,^31^. However, the reflection enhancement of object’s subwavelength information could not compensate its decaying at the 40-nm air distance. Thus, the imaging contrast of 60-nm half-pitch dense-lines is only 0.37 with plasmonic cavity lens shown in Fig. 2c.

Further enhancement of the imaging contrast could be obtained by the wavevector shifting of evanescent wave components under high spatial frequency spectrum OAI, as illustrated in Fig. 3b, which plots the Fourier spectra of the electric field inside the plasmonic cavity lens at the middle plane of the Pr layer. For the NI case (*k*_*x*,*inc*_ = 0), the featured Fourier components of 60-nm half-pitch dense lines are *k*_*x*_ = ±3.0*k*_0_. The NI case does not yield a great enhancement from the plasmonic cavity lens, owing to the small OTF magnitude in Fig. 3a. However, for the high spatial frequency spectrum OAI with *k*_*x*,*inc*_ = 1.5 *k*_0_, the featured Fourier components are shifted to smaller wavevectors as *k*_*x*_ = ±1.5 *k*_0_, delivering a two-fold higher enhancement than the NI case (Fig. 3b). This enhancement gives rise to the imaging contrast approximately 0.85 for the plasmonic cavity lens with a 40-nm air distance, as shown in Fig. 2d. Thus, the cooperation of the plasmonic cavity lens with high spatial frequency spectrum OAI allows for plasmonic lens imaging far beyond near-field diffraction limit.

The plasmonic cavity lens structure could modulate the ratio between the electric field components |*E*_*x*_| and |*E*_*z*_|, as shown in the comparison between Fig. 3c,d, which plays a crucial role in obtaining the high-fidelity image under high spatial frequency spectrum OAI. This point could be well understood from the imaging contribution of the |*E*_*x*_| and |*E*_*z*_| components in the near-field imaging process for L/S = 1 dense lines. As shown in Fig. 3e for the superlens with *k*_*x,inc*_ = 1.5 *k*_0_ illumination, |*E*_*z*_|^2^ becomes the main imaging component and displays a half-period shift compared to |*E*_*x|*_^2^ distribution in the imaging region. This shift occurs mainly because of the *π*/2 shift between *E*_*z*_ and *E*_*x*_ components with similar amplitudes for the wide range of evanescent Fourier components *k*_*x*_ shown in Fig. 3c. For the plasmonic cavity lens, the surface plasmon coupling between two Ag films delivers the damped |*E*_*z*_|^2^ as illustrated in the optical transfer function (OTF) curves (Fig. 3d), and the component |*E*_*x*_|^2^ dominates the imaging area^32^,^33^ with images position fidelity as shown in Fig. 3f,g.

The air working distance elongation could be approximately evaluated by assuming that the magnitude of the transferred Fourier components *k*_g_ in plasmonic cavity lens under NI is compensated by the enhanced magnitude of the shifted Fourier components *k*_*x,inc*_ or *k*_*x,inc*_ − *k*_g_. This compensation yields the air distance in the form


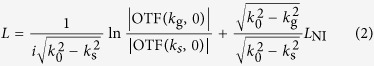


where *L*_NI_ and *L* represent the air distance under NI and high spacial frequency spectrum OAI, respectively; and OTF(*k*_g_,0) and OTF(*k*_s_,0) are the optical transfer function values of plasmonic cavity lens with a zero gap distance and |OTF(*k*_s_,0)| = min{|OTF(*k*_*i*_,0)|, |OTF(*k*_*i*_ − *k*_g_,0)|}. The calculation agrees with the simulated results in a similar tendency, as illustrated by the red dashed line in Fig. 2d.

### Experiment results

**Figure 4:**
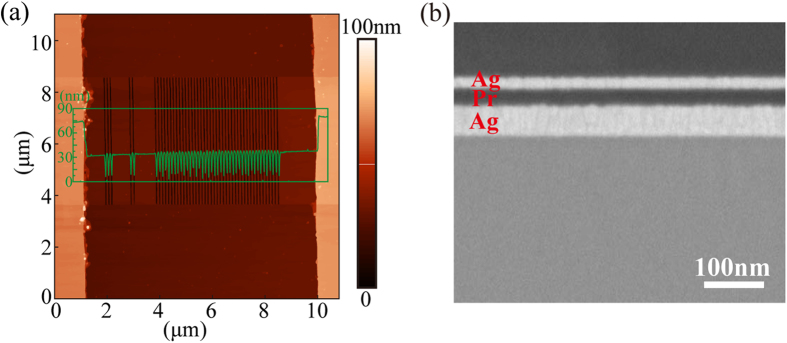
(**a**) AFM image of the Cr mask with a 40-nm air distance and a 60-nm feature-size object. (**b**) SEM image of the Ag-Pr-Ag plasmonic cavity lens structure on the fused silica substrate.

The lithography experiments are performed to demonstrate the effect of imaging beyond the near-field diffraction limit. In Fig. 4a, the AFM-measured profiles of Cr mask with the 40-nm-thick Cr spacer validate the fixed air gap distance in the lithography experiment, in which the plasmonic cavity lens is physical contact with the Cr spacer by air pressure. The cross-sectional SEM image of the plasmonic cavity lens in Fig. 4b shows the smooth Ag interfaces. To reduce negative influence of film roughness on the resolution and resist pattern quality, the films’ surface roughness characterized by the root mean square (RMS) of surface height deviation are carefully controlled with the measured values as 0.43, 0.3 and 0.57 nm for the upper surface of the top Ag layer, Pr layer and bottom Ag layer, respectively.

**Figure 5:**
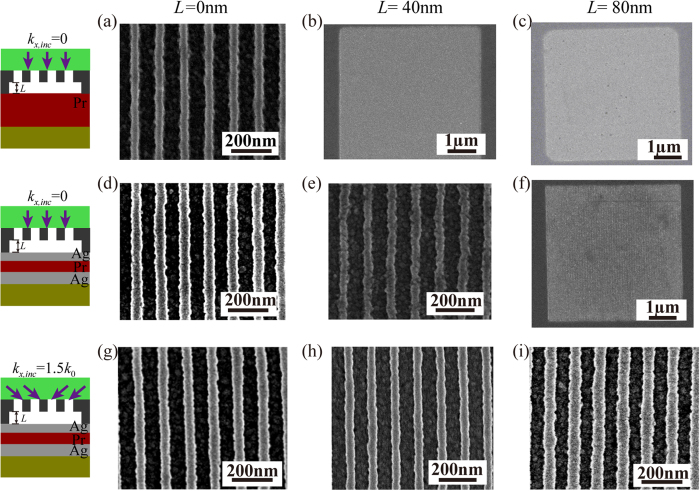
SEM images of resist patterns for 60-nm half-pitch dense lines with variable air distances of 0, 40 and 80 nm. (**a–c**) in the top row are for the near-field lithography under NI; (**d–f**) in the middle row and (**g–i**) in the bottom row represent the plasmonic cavity lens lithography under NI and high spatial frequency spectrum OAI, respectively.

The SEM images of the developed resist patterns for the three schemes are shown in Fig. 5. For the near-field lithography scheme under NI, the maximum air gap distance for the 60-nm half-pitch dense lines is approximately 20 nm, leading to the observation that the recorded images (Fig. 5a–c) could be resolved for a zero gap distance and not be discerned at an air gap distance of 40 nm. Owing to the resonant enhancement of the cavity lens, the air distance in the NI case reaches 40 nm. Accordingly, the scheme in Fig. 5d–f could distinguish the dense lines at an air gap distance of 40 nm but fails at a distance of 80 nm. As high spacial frequency spectrum OAI (*k*_*x,inc*_ = 1.5 *k*_0_) is applied to the plasmonic cavity lens, the Fourier shift effect further enhances the image information and significantly elongates the air distance to 120 nm in Fig. 2e. The recorded resists exhibit well resolved images in Fig. 5g–i under the 80-nm air distance, and a longer distance can probably be achieved.

**Figure 6:**
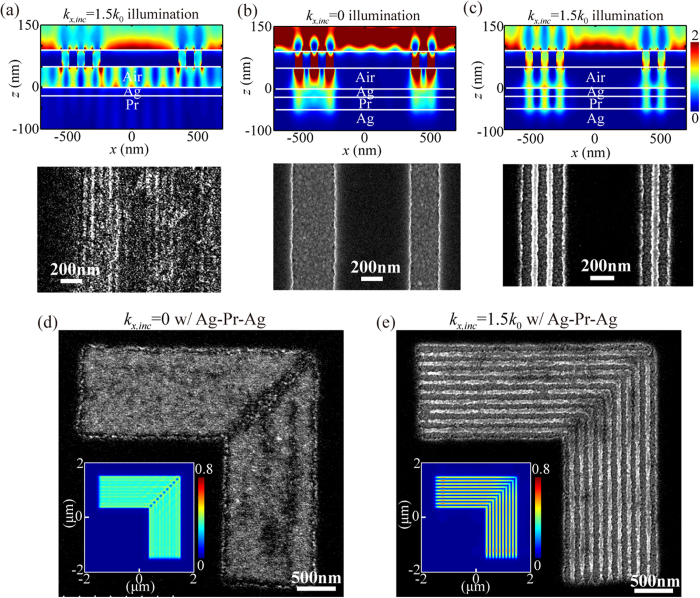
Simulation and experimental imaging results of a variety of nano-patterns for the superlens under high spatial frequency spectrum OAI (a), the plasmonic cavity lens under NI (b) and the high spatial frequency spectrum OAI (c). (**d,e**) are the SEM images of 2D resist patterns for the plasmonic cavity lens with NI and high spatial frequency spectrum OAI, respectively. The insets in (**d,e**) are the simulated images. In all patterns, feature size of mask patterns and air gap distance is fixed as 60 nm and 50 nm, respectively. The other parameters are the same as those in Fig. 1.

Moreover, experimental demonstrations of exceeding the near-field diffraction limit is provided for imaging one-dimensional multi-lines and two-dimensional L-shape dense lines. In the control case of the superlens under high spatial frequency spectrum OAI in Fig. 6a, the simulated images and exposed resist patterns exhibit great aberrations compared with the mask patterns of 60-nm half-pitch multi-lines. This phenomenon can be understood by considering the negative contribution of the longitudinal electric filed component, as shown in Fig. 3e. For the plasmonic cavity lens under NI and a 50-nm air distance, the simulated images in Fig. 6b show low contrast, and the lines are completely blurred in the recorded resist pattern. When plasmonic cavity lens with illumination wavevector *k*_*i*_ = 1.5 *k*_0_ is introduced (Fig. 6c), the imaging patterns of multi-lines could be clearly discriminated with high contrast and fidelity in the simulation and SEM images.

The proposed method could be readily extended to two-dimensional pattern of L-shape dense lines by introducing high spatial frequency spectrum OAI in four directions, with azimuthal angles of *nπ*/2, (*n* = 0, 1, 2, 3). In the control case under NI in Fig. 6d, the L-shape dense-lines with a 60-nm half pitch could not be distinguished at an air distance of 50 nm. As shown in Fig. 6e, the simulation and SEM image of the resist pattern exhibit resolvable images. The widened boundary lines and corner rounding effect observed in Fig. 6c,e exhibit some imaging aberrations and could be relieved by referring to the optical proximity mask correction^20^,^34^.

**Figure 7:**
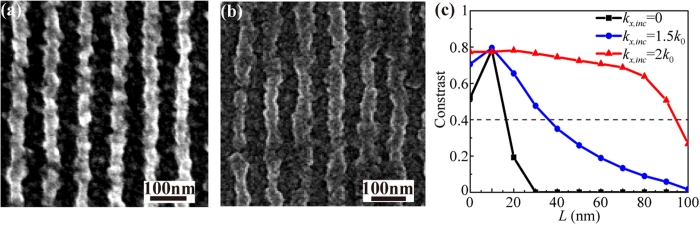
SEM images of resist patterns for 45-nm half-pitch dense-lines by plasmonic cavity lens lithography under high spatial frequency spectrum OAI (*k*_*x,inc*_ = 1.5 *k*_0_) with an air distance of (a) 20 nm and (b) 40 nm. (**c**) Simulated imaging contrast versus the air distance *L* for 45-nm half-pitch dense-lines. The same geometry parameters are shown in Fig. 1.

The experiment of imaging 45-nm half-pitch dense-lines was performed by non-contact plasmonic cavity lens lithography under high spatial frequency spectrum OAI (*k*_*x,inc*_ = 1.5 *k*_0_). Figure 7a,b show that the dense lines could be resolved even at a 40-nm air distance and better image in resist was achieved at an air distance of 20 nm. Figure 7c plots the simulated imaging contrast under light illumination with *k*_*x,inc*_ = 0, 1.5 *k*_0_ and 2 *k*_0_. The air distance could be extended to approximately 90 nm for *k*_*x,inc*_ = 2 *k*_0_, nearly 6 times enhancement in comparison to the *k*_*x,inc*_ = 0 case. This suggests that evanescent wave or plasmonic wave illumination would be capable for deeper subwavelength plasmonic cavity lens lithography far beyond the near-field diffraction limit^35^.

## Discussion

In summary, plasmonic cavity lens incorporating high spatial frequency spectrum OAI is proposed and demonstrated to exceed the near-field diffraction limit associated with the wavelength and distance away from the objects. The experiments show that the 60-nm half-pitch dense lines are clearly discriminated in plasmonic cavity lens lithography using I-line 365-nm wavelength light and high spatial frequency spectrum OAI (*k*_*x,inc*_ = 1.5 *k*_0_) at an air distance of 80 nm. Higher resolution with a 45-nm half pitch is demonstrated in experiments at an air distance of 40 nm, which could be further extended to approximately 90 nm via *k*_*x,inc*_ = 2 *k*_0_ illumination, as illustrated in simulation. As an analogue with the resolution enhancement techniques in conventional projection photolithography^34^,^36^, this investigation validates the OAI for deep subwavelength plasmonic lens imaging far exceed the near-field diffraction limit. The proposed approach will probably lead to potential applications in nanolithography and high-density optical storage, among other fields.

## Methods

### Numerical simulations

Rigorous coupled wave analysis (RCWA) was applied to calculate the imaging contrast distributions shown in Fig. 2 and the transmission amplitude distributions shown in Fig. 3. The RCWA code was written based on the equations in Ref. 37. The electric field intensity distributions shown in Fig. 6 were simulated using the commercially available CST software.

### Fabrication procedures for the plasmonic cavity lens and mask

For the Ag-Pr-Ag plasmonic cavity lens structure, the 50-nm-thick Ag film was first deposited onto a fused silica substrate via the thermal evaporation at a base pressure of 5.0 × 10^−4^ Pa with a deposition rate of 5 nm/s. An approximately 30-nm-thick photoresist of the diluted AR-P3170 (diluted by ALLRESIST GMBH, Strausberg, 30 nm @ 4000 rpm) was spun onto the substrate to record the near-field images. After prebaking the photoresist on a hotplate at 100 °C for 5 min, a 20-nm-thick Ag film was evaporated on the photoresist (base pressure of 5.0 × 10^−4^ Pa, deposition rate of 1 nm/s).

The fabrication of the Cr mask began with the deposition of the Cr film via Magnetron Sputtering (RF power of 400 W, deposition rate of 0.5 nm/s and temperature of 300 °C) onto a sapphire substrate. The thickness of the deposited Cr film was equal to the expected air gap distance. Next, a 9-μm-wide transparent window on the Cr film was made via UV photolithography and wet etching. Subsequently, the second deposition of another 40-nm Cr film was applied to achieve the Cr layer of mask pattern. Finally, the nano-pattern object was milled onto the Cr layer in the window region by a focused ion beam instrument (Helios Nanolab 650, FEI Company, @30 kV Accelerating Voltage).

### Exposure and Development of the Lithography Experiments

The substrate with the Ag-Pr-Ag plasmonic cavity lens was in physical contact with the Cr mask with the aid of air pressure (0.3 MPa). The high spatial frequency spectrum OAI (*k*_*x,inc*_ = 1.5 *k*_0_) was generated by impinging two uniform and incoherent UV light beams from an I-line mercury lamp exposure source into a sapphire prism, which was put in close contact with sapphire substrate of the masks via a refractive index matching liquid. The two UV lights are configured with a divergence of approximately ±3° and a line width of ±5 nm. The exposure with a flux of 1.5 mW/cm^2^ and an exposure time ranging from 10 to 30 s was applied in the experiment. Before the developing process, the top Ag layer was physically peeled off. The photoresist was developed by the diluted AR 300-35 (ALLRESIST GMBH, Strausberg) with de-ionized water at the ratio of 1:1 for the period of 40 s.

## Additional Information

**How to cite this article**: Zhao, Z. *et al.* Going far beyond the near-field diffraction limit via plasmonic cavity lens with high spatial frequency spectrum off-axis illumination. *Sci. Rep.* **5**, 15320; doi: 10.1038/srep15320 (2015).
